# Lenvatinib induces anticancer activity in gallbladder cancer by targeting AKT

**DOI:** 10.7150/jca.50292

**Published:** 2021-04-24

**Authors:** Jianwen Ye, Lei Qi, Jialu Liang, Ke Zong, Wentao Liu, Renfeng Li, Ruo Feng, Wenlong Zhai

**Affiliations:** 1 Department of Hepatobiliary and Pancreatic Surgery, The First Affiliated Hospital of Zhengzhou University, Zhengzhou, Henan 450052, P.R. China.; 2 Key Lab of Digestive Organ Transplantation of Henan Province, Open and Key Laboratory of Hepatobiliary and Pancreatic Surgery and Digestive Organ Transplantation at Henan Universities, Zhengzhou Key Laboratory of Hepatobiliary and Pancreatic Disease and Organ Transplantation, Zhengzhou, Henan 450052, P.R. China.; 3 Department of Pharmacy, The First Affiliated Hospital of Zhengzhou University, Zhengzhou, Henan 450052, P.R. China.; 4 Department of Histology and Embryology, Medical College of Zhengzhou University, Zhengzhou 450052, P.R. China.

**Keywords:** lenvatinib, gallbladder cancer, proliferation, apoptosis, migration, AKT

## Abstract

Gallbladder cancer (GBC) is characterized by poor prognosis, early metastasis, and high recurrence rates, which seriously threaten human health. The effect of lenvatinib, a widely used drug in anti-hepatocellular carcinoma in China, on GBC progress, as well as its underlying molecular mechanism, remains unclear. Therefore, the present study investigated the effect of lenvatinib on human GBC GBC-SD and NOZ cells and its underlying mechanisms. A series of experiments, including cell proliferation, clone formation, wound healing, and cell migration and invasion assays, as well as flow cytometry, were performed to investigate the anticancer effect of lenvatinib on GBC. Western blotting was used to detect alterations in protein expression of CKD2, CKD4, cyclin D1, caspase-9, matrix metalloproteinase (MMP)-2, cell migration-inducing protein (CEMIP) and phospho-AKT (p-AKT). In addition, the chemosensitivity of lenvatinib-treated GBC cells to gemcitabine (GEM) and whether the activation of phosphoinositide 3 kinase (PI3K)/AKT contributed to the chemoresistance were determined. Finally, the anticancer effect of lenvatinib *in vivo* was detected using a xenograft mouse model. These data showed that treatment with lenvatinib inhibited cell proliferation, colony formation ability, migration, induced apoptosis, regulated cell cycle and resulted in decreased resistance to GEM. Treatment with lenvatinib decreased the expression of MMP-2, CEMIP, CDK2, CDK4 and cyclin D1, and increased the expression of cleaved caspase-9, which was mediated by the inactivation of the PI3K/AKT pathway *in vitro.* In addition, lenvatinib inhibited autophagy in GBC-SD and NOZ cells. Besides, Lenvatinib suppressed GBC cell growth *in vivo* by targeting p-AKT. In combination, the present data indicated that lenvatinib plays a potential anticancer role in GBC by downregulating the expression of p-AKT.

## Introduction

Gallbladder cancer (GBC), one of the most common malignant tumors of the digestive system and has an overall 5-year survival rate of <5% 1. Currently, surgical resection is the most effective treatment for GBC, but its recurrence and mortality rates remain high 2. Other treatment options for advanced GBC include chemotherapy and radiotherapy. However, the therapeutic efficacy of those are limited and the prognosis of advanced GBC patients still very poor. It is therefore necessary to develop new treatments for advanced GBC patients.

In the past decade, sorafenib, multi-kinase inhibitor [against vascular endothelial growth factor receptor (VEGFR)1-3, fms-like tyrosine kinase 3, RET and RAF kinases, platelet-derived growth factor (PDGF) and KIT], has crucially contributed to the systemic therapy of patients with advanced or metastatic hepatocellular carcinoma (HCC) or clear cell carcinoma of the kidney 3, 4. However, the overall response rate of sorafenib in the treatment of advanced HCC is not high (only 2.3%), the improvement of patients' main symptoms is not obvious, the side effects are obvious and there is a lack of effective molecular markers for prediction, which, combined with its high price, limit its use in patients with advanced HCC 5. As a result, several clinical trials on molecularly-targeted drugs, such as sunitinib 6 and brivanib 7, emerged but failed. Recently, lenvatinib has become another Food and Drug Administration-approved molecularly-targeted drug for the first-line treatment of advanced or metastatic hepatocellular carcinoma. In the REFLECT clinical trial, 954 HCC patients were randomly divided into the lenvatinib and sorafenib groups. As shown in that study, lenvatinib has been shown to obtain satisfactory results in increasing the OS, progression-free survival (PFS) and TTP, as compared with sorafenib in unresectable HCC patients 5. In addition, the subgroup analysis of the REFLECT study demonstrated that the efficacy of lenvatinib in patients with advanced HCC in China was more significant than in that in the global population, which suggested that lenvatinib can serve as another alternative first-line targeted drug for advanced HCC.

Lenvatinib, another new oral, molecularly-targeted anti-cancer drug, possesses an anti-cancer effect in HCC by inhibiting multi-targeted tyrosine kinase inhibitors against VEGFR1-3, fibroblast growth factor receptor (FGFR)1-4, PDGFRα, RET and KIT 8. Previous studies have shown that lenvatinib suppressed HCC and anaplastic thyroid cancer (ATC) cell growth *in vitro* and *in vivo* 9, 10. In addition, treatment with lenvatinib and I-131 significantly inhibited cell growth and induced apoptosis by activating endoplasmic reticulum-associated apoptosis mechanisms 11. Heptanomide-lenvatinib combination treatment inhibited drug resistance by inhibiting the FGFR signaling pathway in ATC cancer stem cells 12. Although the anti-cancer effect of lenvatinib against HCC and ATC have been reported, both the effect and the underlying molecular mechanism of lenvatinib are yet to be investigated in GBC cells.

The phosphoinositide 3 kinase (PI3K)/AKT pathway is one of the most important signaling pathways that plays a key role in cell biological behavior, including cell growth, apoptosis, invasion, differentiation and metabolism 13. Increasing evidence has shown that the PI3K/AKT pathway is upregulated and involved in GBC 14, 15. It is also known to play an important role in imparting chemoresistance, and its activation has been shown to increase the chemoresistance of cancer cells 16, 17. A previous study reported that the expression of PI3K/AKT was increased in sorafenib-resistant liver cancer cells, and that the inactivation of AKT can sensitize cells to sorafenib-mediated apoptosis 18. However, the role of lenvatinib in the activation of PI3K/AKT remains unclear.

In the present study, in order to further investigate the role of lenvatinib in cell biological behavior, as well as the PI3K/AKT pathway in GBC cells, *in vitro* and *in vivo* assays were performed. The data from this study demonstrated that lenvatinib inhibited cell growth, colony formation, migration and invasion, and induced cell apoptosis and regulated cell cycle arrest progression. Besides, we first reported that treatment with lenvatinib significantly suppressed autophagy. In addition, treatment with lenvatinib enhanced the sensitivity of GBC to gemcitabine (GEM) by downregulating the activation of the PI3K/AKT pathway. The present findings suggested that lenvatinib treatment may have a therapeutic potential in patients with GBC.

## Materials and methods

### Cell culture

The human GBC GBC-SD and NOZ cell lines were obtained from Procell Life Science & Technology Co., Ltd. The GBC-SD cells were cultured in Rosewell Park Memorial Institute (RPMI)-1640 medium (cat. no. 31800; Beijing Science & Technology Co., Ltd.) and NOZ were cultured in DMEM/F12 (cat. no. SH30023.01; HyClone; GE Healthcare Life Sciences) supplemented with 10% fetal bovine serum (FBS; Gemini Bio Products) at 37 °C in a humidified atmosphere with 5% CO_2_.

### Cell viability assay

Lenvatinib was purchased from Selleck Chemicals (cat. no. S5240) and dissolved in dimethyl sulfoxide. Cell viability was determined using Cell Counting Kit-8 (CCK-8; Dojindo Molecular Technologies, Inc.) assay. Human GBC-SD and NOZ cells were seeded onto 96-well plates at a density of 5×10^3^ cells/well. After 24 h, the medium was removed and replaced with medium containing 0, 10, 20, 40 or 80 µM lenvatinib for different times (0, 24, 48 and 72 h). Following treatment, the cells were incubated with 10 μl CCK-8 solution in a 5% CO_2_ incubator at 37 °C for 1 h. The OD values were then obtained by measuring the absorbance at a wavelength of 450 nm using a micro-plate reader (Thermo Fisher Scientific, Inc.).

### Colony formation assay

GBC-SD and NOZ cells were seeded onto 6-well plates at a density of 5×10^2^ cells/well and culture medium containing lenvatinib was replaced every 3 days and incubated for 2 weeks until clones were visible. Next, cells were washed with PBS and fixed with methanol for 20 min, following staining with 0.1% crystal violet (Institute of Biotechnology) for 30 min at room temperature. Colonies containing >50 cells were counted under an inverted microscope. (IX71; Olympus Corporation).

### Flow cytometry

For cell apoptosis analysis, GBC-SD and NOZ cells were detected using an Annexin V/fluorescein isothiocyanate (FITC) Apoptosis Detection kit (BD Biosciences), according to the manufacturer's instructions. Human GBC-SD and NOZ cells were harvested following treatment with 25 and 50 µM lenvatinib for 48 h. The cell suspension was transferred to a culture tube and 5 µl propidium iodide (PI) fluorescent dye was added. Cells were then incubated at room temperature for 15 min in the dark, and 400 µl binding buffer was then added. Flow cytometry results were analyzed using a FACS Calibur system (BD Biosciences). For cell cycle arrest analysis, human GBC-SD and NOZ cells were collected following treatment with lenvatinib, washed with cold PBS and fixed in cold 75% ethanol at 4 °C overnight. Subsequently, GBC-SD and NOZ cells were washed with cold PBS, incubated with RNase and stained with PI in the dark for 15 min. The proportion of cells at the G0/G1, S and G2/M phases was determined using the FACS Calibur system, according to the manufacturer's instructions. Data were analyzed by BD FACS Diva 8.0.1 software.

### Wound healing assay

Human GBC‑SD and NOZ cells were cultured in 6‑well plates overnight at 37 °C. Once the GBC‑SD and NOZ cells were spread over the plates, a vertical long wound was scratched into the cells using a 200-μl pipette tip. Following this, the scratched cells were washed twice with PBS and images of the cells were captured using an inverted microscope (IX71; Olympus Corporation). The cells were treated with 25 μM lenvatinib and cultured in medium for 48 h at 37 °C, following which a further image was obtained using an inverted microscope. The wound closure ratio was calculated as follows: (0 h width‑48 h wound width)/0 h wound width.

### Migration and invasion assay

For the migration assay, GBC‑SD and NOZ cells were suspended in 100 μl serum‑free RPMI-1640 medium and placed in the upper chamber of a Transwell chamber coated with (for the invasion assay) or without (for the migration assay) Matrigel (BD Biosciences). In addition, 600 µl medium containing 10%FBS was added in the lower chamber of a Transwell. Subsequently, 25 or 50 μM lenvatinib was applied to the chamber and incubated for 24 h at 37 °C. Invasive cells on the lower surface of the membrane were washed with PBS and fixed in methanol for 20 min. The invasive cells were stained with 0.1% crystal violet for 20 min at room temperature. The cellular migration or invasion through the membrane was visualized using an inverted microscope (IX71; Olympus Corporation).

### Western blotting

Cells were collected and total protein was extracted from GBC-SD and NOZ cells on ice using RIPA lysis buffer (Beyotime Institute of Biotechnology) with a protease inhibitor cocktail (104 mM AEBSF, 80 μM Aprotinin, 5 mM Bestatin, 1.5 mM E-64, 2 mM Leupeptin and 1.5 mM Pepstatin A; MedChemExpress) for 20 min. The protein concentration was determined using a BCA-kit (Beyotime Institute of Biotechnology). Equal amounts of protein were separated by SDS-PAGE and transferred to PVDF membranes (Millpore,cat.no IPVH00010). The membranes were immunoblotted with the following primary antibodies: Matrix metalloproteinase (MMP)-2 (dilution, 1:800; cat. no., 10737-2-AP), cell migration-inducing and hyaluronan-binding protein (dilution, 1:600; cat. no., 21129-1-AP), CDK2 (dilution, 1:2,000; cat. no., 10122-1-AP), CDK4 (dilution, 1:1,000; cat. no., 11026-1-AP), Cyclin D1 (dilution, 1:5,000; cat. no., 60186-1-Ig), Caspase-9 (dilution, 1:600; cat. no., 66169-1-Ig) and β-actin (dilution, 1:5,000; cat. no., 60008-1-Ig) (all were purchased from ProteinTech, China), and p-AKT (dilution, 1:1,000; cat. no., 4060; Cell Signaling Technology, Inc.) at 4 °C overnight. Following incubation with a horseradish peroxidase-conjugated goat anti-rabbit IgG (dilution, 1:5,000; cat. no., IH-0011) or anti-mouse IgG antibodies (dilution, 1:5,000; cat. no., IH-0031; both from DingGuo BioTech Co., Ltd.) for 1 h at room temperature, the proteins were visualized using an enhanced chemiluminescence western blotting detection kit (Thermo Fisher Scientific, Inc.), according to the manufacturer's instructions. β-Actin or GAPDH served as the internal control.

### Tumor xenograft mouse models

Eighteen female immunodeficient BALB/c nude mice (age, 4-6 weeks; weight, 16±2 g) were purchased from Vital River Laboratory Animal Technology Company Limited. All mice were raised under pathogen‑free conditions and maintained in a controlled environment (temperature, 25±2 °C; relative humidity, 70±5%; 12-h light/dark cycle) and fed standard laboratory food and water. The tumor xenografts were generated by a subcutaneous injection of GBC-SD cells (5×10^7^/ml × 125 μl) into the right flank of female nude mice. The mice were randomly divided into three groups of 6 mice: The control and the lenvatinib groups, administered with 15 or 30 mg/kg (intraperitoneal injection, daily) 9, 19. Tumor size and weight were measured using calipers and electronic balance every 2 or 3 days. The tumor volume was determined according to the following formula: Tumor volume (mm^3^) = 0.5× (length × width^2^). The maximum tumor diameter in the present study was 15.74 mm, which was allowable by ethical guidelines. After 22 days, the animals were anesthetized with 1% pentobarbital (50 mg/kg) and sacrificed by breaking their neck. The tumor tissues were removed and measured. Xenograft tumors were collected and fixed in 10% formalin for 24 h at room temperature, following which they were paraffin-embedded and cut into 4-μm sections for immunohistochemical analysis.

### Immunohistochemistry

The slides were incubated in heated antigen retrieval solution (10 mmol/l citrate buffer, pH 6.0), and subsequently treated with 3% hydrogen peroxide for 10 min at room temperature to block endogenous peroxidases. Following washing with PBS, the slides were incubated with a diluted primary antibody p-AKT (dilution, 1:100; cat. no., 4060; Cell Signaling Technology, Inc.) at 4 °C overnight and then with the secondary antibody (1:500 dilution; cat. no., PV-9000; ZSGB-BIO) for 20 min at room temperature. The reaction was developed using a 3,3'‑diaminobenzidine kit (1:50 dilution in buffer; cat. no., ZLI-9017; ZSGB-BIO). Finally, hematoxylin was applied to counterstain the nuclei prior to dehydration and mounting. The stained slides were observed under a light microscope (CX31; Olympus Corporation; original magnification, ×200).

### Statistical analysis

All data are presented as the mean ± standard deviation using SPSS 17.0 software (SPSS Inc.) from at least three independent experiments. Statistical differences between groups were determined by Student's t-test followed by Shapiro-Wilk W test, or one-way ANOVA followed by Bonferroni test. P<0.05 was considered to indicate a statistically significant difference.

## Results

### Lenvatinib suppressed the viability and colony formation of GBC-SD and NOZ cells

**Figure 1:**
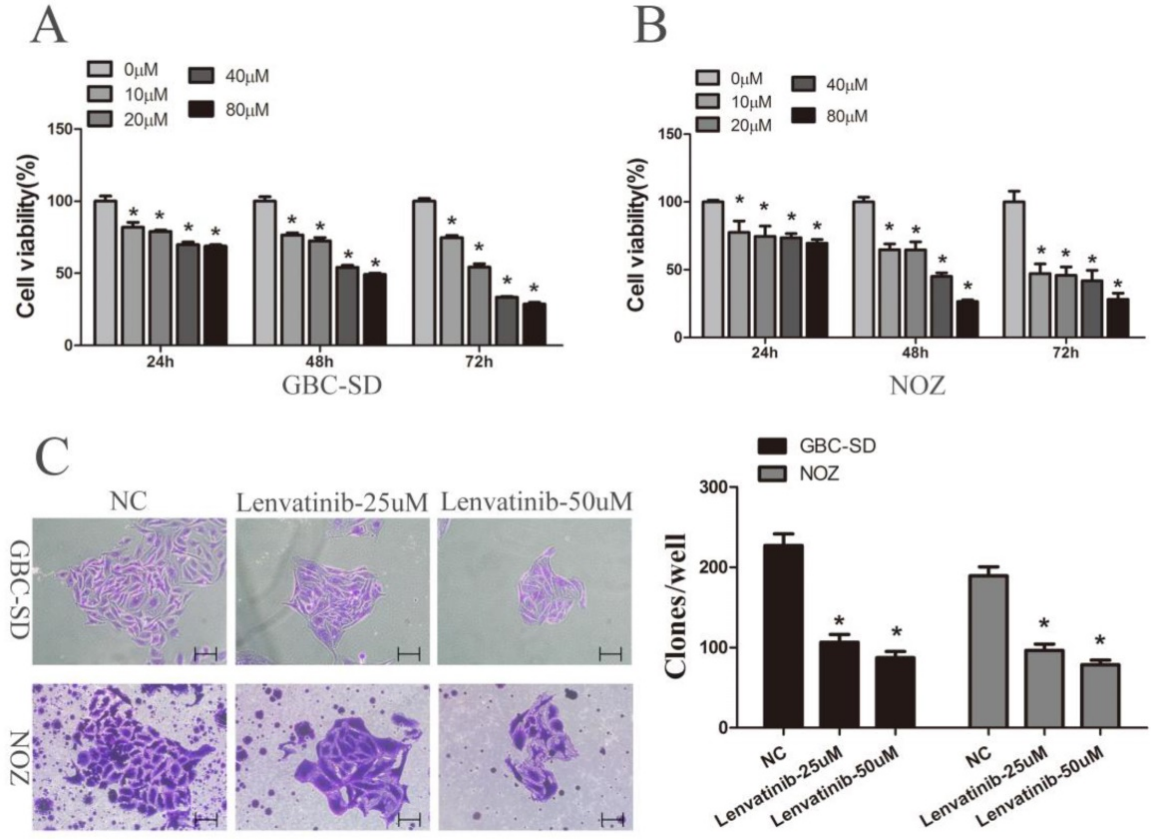
Lenvatinib inhibited proliferation and colony formation in GBC-SD and NOZ cells. GBC-SD and NOZ cells were treated with different concentrations of lenvatinib for 24, 48 and 72 h. **(A and B)** Cell viability was examined by Cell Counting Kit-8 assay. **(C)** Colony formation. ^*^P<0.05 vs. 0 µM. NC, negative control.

To determine the cytotoxicity of lenvatinib, GBC-SD and NOZ cells were exposed to different concentrations of lenvatinib (0-80 µM) for different time intervals (0, 24, 48 and 72 h). A CCK-8 assay was then carried out to detect the effects of lenvatinib on GBC-SD and NOZ cells. Data from Fig. 1A and 1B showed that cellular growth was significantly suppressed by lenvatinib in a dose- and time-dependent manner. The IC50 values of lenvatinib in GBC-SD and NOZ cells at 24, 48 and 72 h were 155.275, 69.659 and 33.403 µM; 8618.871, 28.642 and 10.035 µM, respectively, and the concentrations of 25 and 50 µM for 48 h were chosen for the following study. The effect of lenvatinib on the colony formation ability of GBC-SD and NOZ cells was also observed. As shown in Fig. 1C, treatment with lenvatinib exhibited a significant decrease in the colony number, when compared with the negative control (NC) group. These data demonstrated that treatment with lenvatinib effectively inhibited cell viability and colony formation in GBC-SD and NOZ cells.

### Lenvatinib induced cell apoptosis and inhibited cell cycle arrest in GBC-SD and NOZ cells

**Figure 2:**
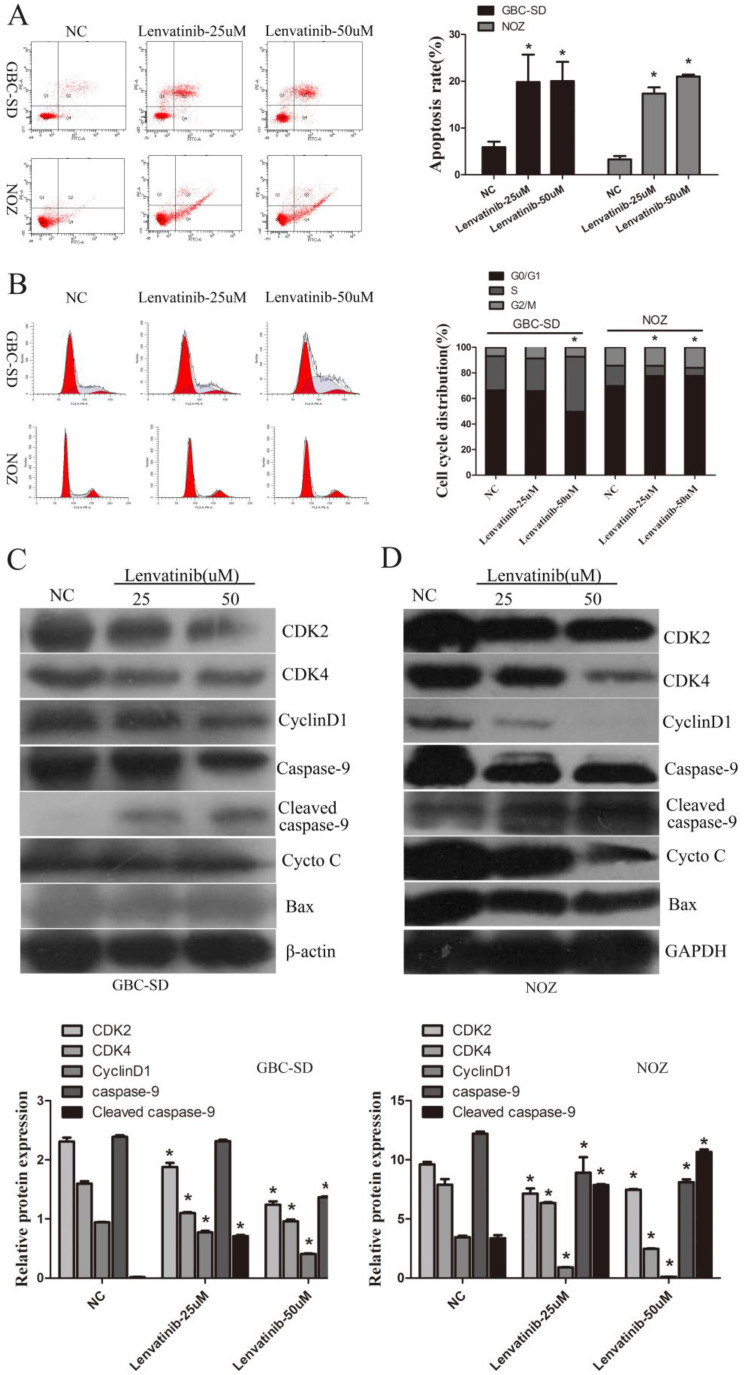
Lenvatinib induced apoptosis and regulated cell cycle arrest progression in GBC-SD and NOZ cells. GBC-SD and NOZ cells were treated with 25 or 50 µM lenvatinib for 48 h, stained with Annexin V-FITC and PI, and analyzed by flow cytometry. **(A)** Quantification of apoptotic rate. **(B)** Distribution of cell cycle. **(C and D)** Protein expression of caspase-9, CytoC, Bax, CDK2, CDK4 and cyclin D1 were detected by western blotting. β-actin or GAPDH was used as a loading control. ^*^P<0.05 vs. NC. Len, Lenvatinib; FITC, fluorescein isothiocyanate; CytoC, cytochrome *c*; Bax, Bcl-2-associated X protein; NC, negative control.

To detect the effect of lenvatinib on cell apoptosis, GBC-SD and NOZ cells were stained with Annexin V-FITC/PI and quantified by flow cytometry. As shown in Fig. 2A, the GBC-SD and NOZ cell apoptotic rate of the NC and 25 and 50 µM lenvatinib treatment groups treated for 48 h was 5.9±1.1, 19.8±5.8, and 20.3±4.1%; 3.3±0.7, 17.3±1.3, and 21.0±0.4%, respectively. These data suggested that treatment with lenvatinib significantly promoted cell apoptosis in GBC-SD and NOZ cells. Furthermore, as shown in Fig. 2B, treatment with 50 µM lenvatinib also inhibited cell cycle progression by inducing G0/G1 phase arrest in GBC-SD cells. While treatment with 25 or 50 µM lenvatinib also suppressed cell cycle progression by inducing S phase arrest in NOZ cells. In addition, the expression of caspase-9, cytochrome *c* (CytoC), Bcl-2-associated X protein (Bax) and relative cell cycle proteins CDK2, CDK4 and cyclin D1 were investigated by Western blotting to explore the mechanism of lenvatinib-induced apoptosis and inhibited cell cycle. As shown in Fig. 2C and 2D, following the culture of 25 and 50 µM lenvatinib-treated GBC-SD and NOZ cells for 48 h, a significant increase was observed in the expression of cleaved caspase-9. The expression of caspase-9, CDK2, CDK4 and cyclin D1 was decreased, while that of CytoC and Bax exhibited no significant changes in GBC-SD cells and Bax exhibited no significant changes in NOZ cells. These data indicated that treatment with lenvatinib significantly induced cell apoptosis and regulated cell cycle in GBC-SD and NOZ cells by regulating the expression of caspase-9, CDK2, CDK4 and cyclin D1.

### Lenvatinib inhibited the migration and invasion of GBC-SD and NOZ cells

**Figure 3:**
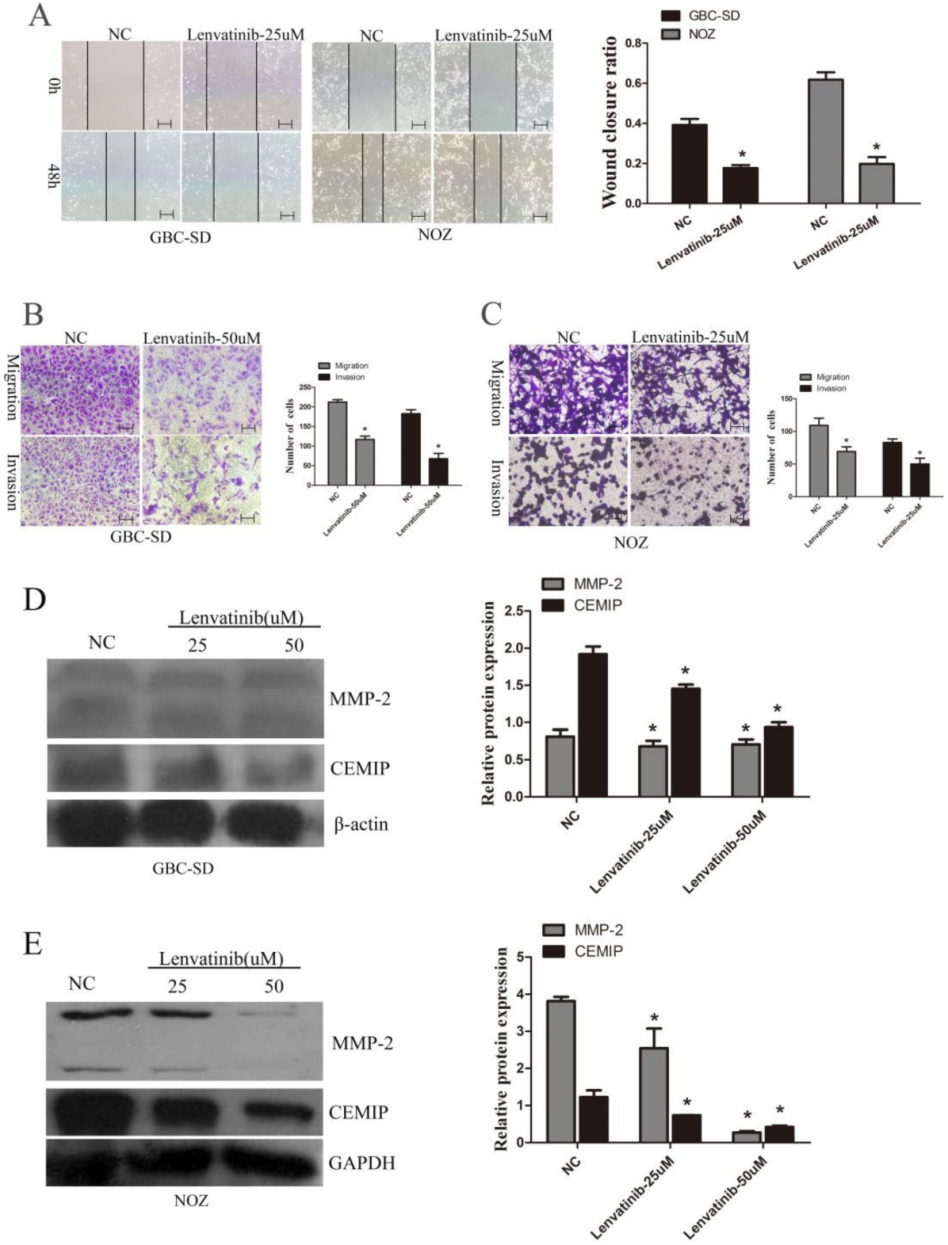
Lenvatinib inhibits migration and invasion in GBC-SD and NOZ cells by downregulating MMP-2 and CEMIP. GBC-SD and NOZ cells were treated with 25 or 50 µM lenvatinib for 48 h. **(A)** Influence of lenvatinib on cell migration by wound closure. **(B and C)** Influence of lenvatinib on cell migration and invasion by Transwell assays. **(D and E)** Analysis of MMP-2 and CEMIP following treatment with lenvatinib in GBC‑SD and NOZ cells, as assessed by western blotting. β-actin or GAPDH was used as a loading control. Original magnification, x100. ^*^P<0.05 vs. NC. CEMIP, cell migration-inducing protein; Len, lenvatinib. MMP, matrix metalloproteinase; NC, negative control.

To further observe the effect of lenvatinib on GBC-SD and NOZ cell migration and invasion, wound healing and Transwell assays were performed to detect the migration and invasion ability of GBC-SD and NOZ cells. As shown in Fig. 3A, the wound closure ratio in GBC-SD cells and NOZ cells in the NC and lenvatinib groups for 48 h were 0.392±0.030 and 0.177±0.014; 0.618±0.037 and 0.198±0.033, respectively. It is also shown in Fig. 3B that the number of migrated and invasive cells in GBC-SD cells per high-power field in the NC and lenvatinib groups were 212.4±5.9 and 116.8±8.7; 182.7±10.1 and 67.7±13.8, respectively. Furthermore, consistent with the above results, the number of migrated and invasive cells in NOZ cells per high-power field in the NC and lenvatinib groups were 109.2±10.9 and 69.0±7.3; 83.0±5.4 and 50.0±8.9, respectively (Fig. 3C). To further elucidate the molecular mechanism involved in cell migration and invasion, the protein expression of MMP-2 and CEMIP was detected by western blotting. As shown in Fig. 3D and 3E, the expression of MMP-2 and CEMIP was decreased following treatment with 25 or 50 µM lenvatinib for 48 h. These results indicated that treatment with lenvatinib may significantly reduce the migration and invasion ability of the cells by inhibiting MMP-2 and CEMIP.

### Lenvatinib suppressed autophagy of GBC-SD and NOZ cells

**Figure 4:**
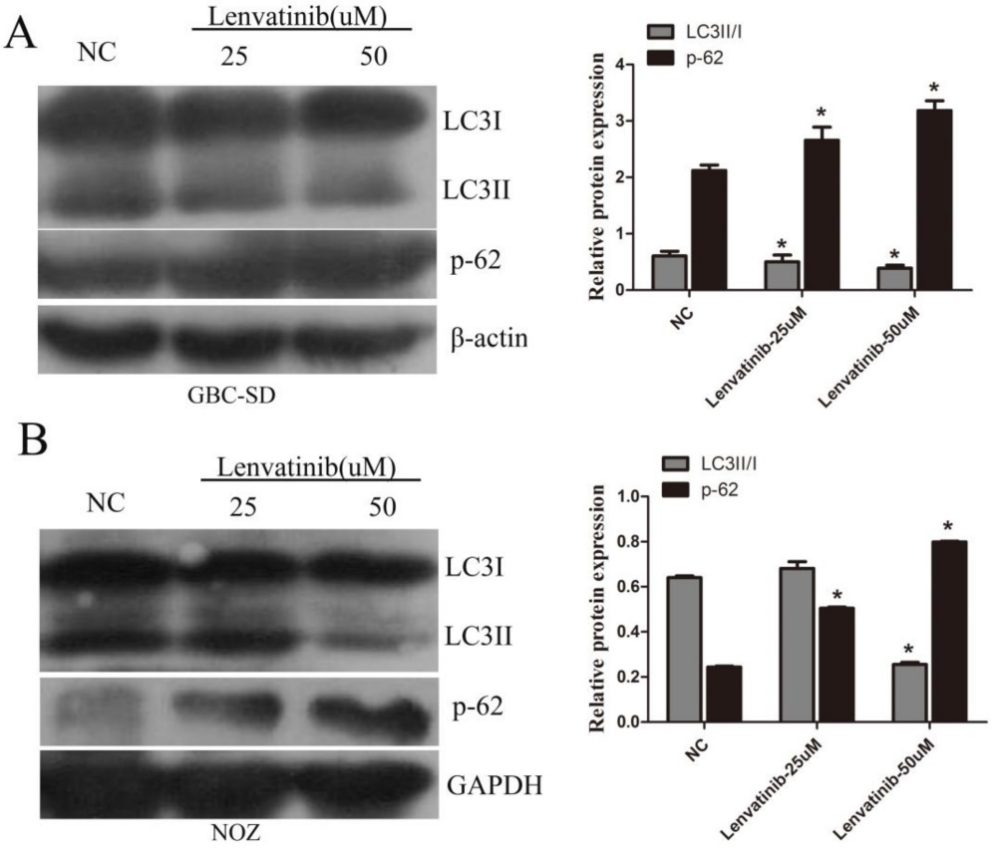
Lenvatinib suppressed autophagy in GBC-SD and NOZ cells. GBC-SD and NOZ cells were treated with 25 or 50 µM lenvatinib for 48 h. **(A and B)** Analysis of LC3II/I ration and p-62 following treatment with lenvatinib in GBC‑SD and NOZ cells, as assessed by western blotting. β-actin or GAPDH was used as a loading control. ^*^P<0.05 vs. NC. LC3, microtubules associated proein 1 light chain 3β; NC, negative control.

GBC-SD and NOZ cells were treated with 25 µM and 50 µM lenvatinib for 48 h. At the end of treatment, cells were harvested and autophagy relative genes were observed by western blot analysis. It was found that treatment with lenvatinib significantly decreased LC3II/I ratio, whereas the expression of p-62 was increased (Fig. 4A and 4B). These results suggested that treatment with lenvatinib inhibited autophagy.

### Lenvatinib suppressed GBC cell growth *in vivo* by targeting p-AKT

**Figure 5:**
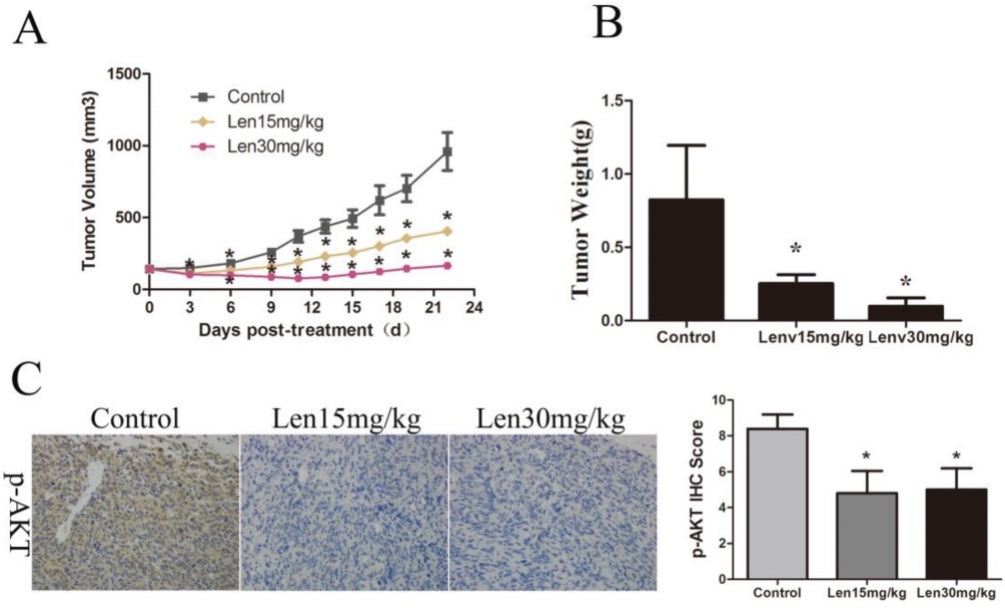
Lenvatinib suppresses tumor growth in GBC‑SD xenografts.** (A)** Growth curves are representative of tumor volumes in BALB/c nude mice in the NC or lenvatinib groups. **(B)** Tumor weight was detected in the NC and lenvatinib groups at the endpoint of the animal experiment. **(C)** The protein expression of p-AKT in GBC-SD tumor tissues was measured by immunohistochemistry. ^*^P<0.05 vs. NC. Len, lenvatinib; NC, negative control; IHC, immunohistochemistry.

To further explore the effects of lenvatinib on GBC, GBC-SD tumor cells were established using a xenograft mouse model. As shown in Fig. 5A and 5B, the tumor volume and weight in the NC group at the end of the treatments were 959.97±322.63 mm^3^ and 0.82±0.37 g, whereas those in the group treated with 15 and 30 mg/kg/day lenvatinib were 404.90±48.81 mm^3^ and 0.25±0.06 g, and 165.69±65.02 mm^3^ and 0.10±0.06 g, respectively. These data showed a marked, dose-dependent decrease in the tumor growth rate and weight of the lenvatinib treatment group. In addition, the protein expression of p-AKT in tumor tissues was also determined by immunohistochemistry. As shown in Fig. 5C, the expression levels of p-AKT were significantly increased in the tumor tissues from the lenvatinib treatment group, as compared with the control group.

### Lenvatinib inhibited the expression of p-AKT and enhanced the sensitivity of GBC-SD and NOZ cells to gemcitabine by inhibiting the expression of p-AKT

**Figure 6:**
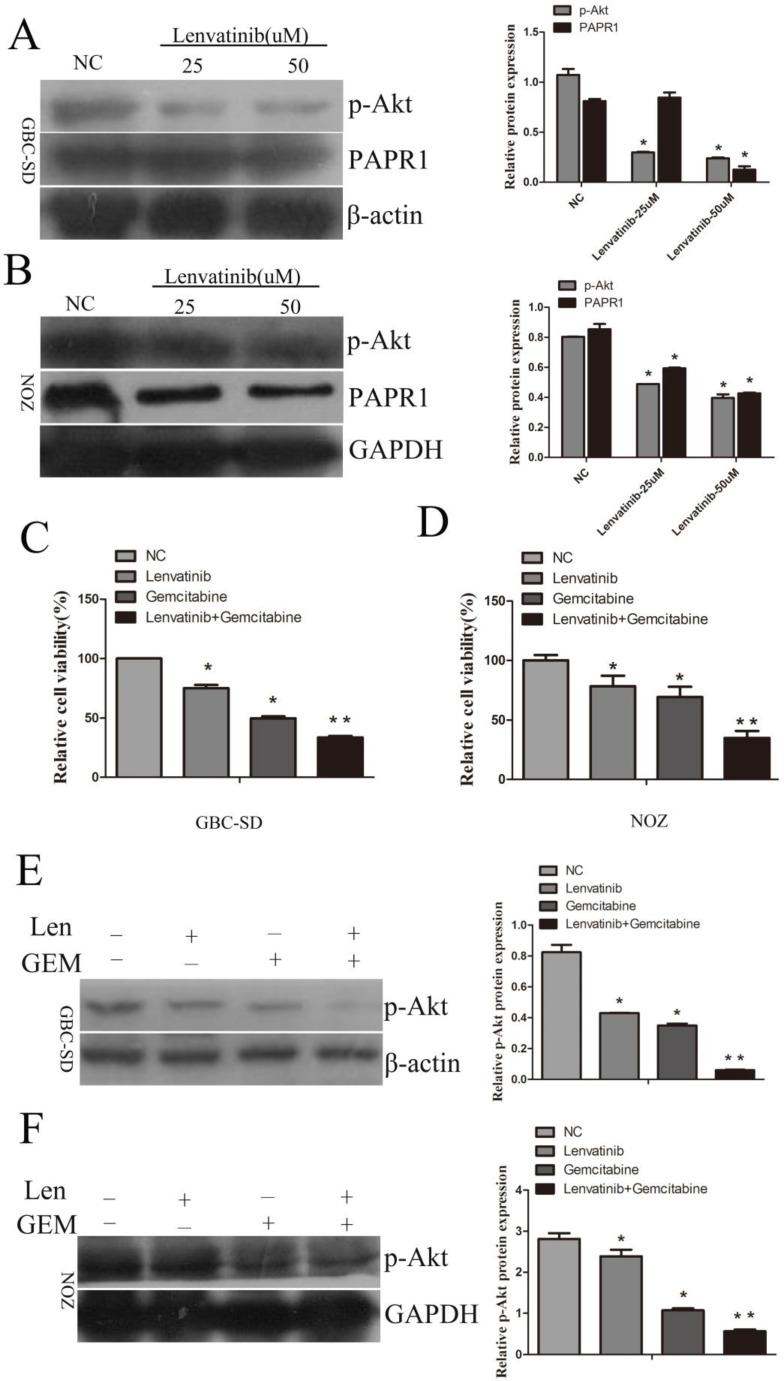
Lenvatinib inhibits the expression of p-AKT and enhances GEM-mediated cell growth inhibition in GBC-SD and NOZ cells by inactivating the expression of p-AKT. GBC-SD and NOZ cells were pretreated with lenvatinib for 1 h, then treated with or without lenvatinib for 48 h. **(A and B)** The protein expression levels of p-AKT and PAPR1 were detected by western blotting with β-actin or GAPDH as a loading control. **(C and D)** Cell growth proliferation was detected by CCK-8. **(E and F)** The protein expression levels of p-AKT were measured by western blotting in GBC-SD and NOZ cells following treatment with lenvatinib and GEM. ^*^P<0.05 vs. NC. ^**^P<0.05 vs. GEM group. NC, negative control; GEM, gemcitabine; PAPR1, poly ADP-ribose polymerase 1; Len, lenvatinib.

To investigate the underlying molecular mechanism of the anticancer effect of lenvatinib, the expression of p-AKT and poly ADP-ribose polymerase 1 (PAPR1) was detected by Western blotting. As shown in Fig. 6A and 6B, the expression of p-AKT was decreased following treatment with 25uM or 50uM lenvatinib in GBC-SD and NOZ cells, whereas no significant changes in the expression of PAPR1 were observed following treatment with 25 µM lenvatinib in GBC-SD cells. To further identify the anticancer effect of lenvatinib on GEM-mediated cell growth inhibition, cell proliferation was detected by CCK-8 assay in GBC-SD and NOZ cells treated with lenvatinib and GEM. As shown in Fig. 6C and 6D, treatment with lenvatinib enhanced the sensitivity of GBC cells to GEM. To further investigate the underlying molecular mechanism of lenvatinib in enhancing the sensitivity of GBC to GEM, the activation of the PI3K/AKT pathway was also examined. As expected, the expression of p-AKT was suppressed following treatment with lenvatinib or GEM (Fig. 6E and 6F). Combined treatment with lenvatinib and GEM significantly inhibited the expression of p-AKT, as compared with treatment with lenvatinib or GEM alone.

## Discussion

GBC is one of the most common types of bile duct cancer worldwide with high recurrence and metastasis rates, despite complete surgical resection. More than 70% of GBC patients are considered to be unsuitable for surgical treatment. The prognosis of GBC is poor and a severe threat to human health and longevity. GEM is currently the first-line anticancer drug for GBC patients, which acts by suppressing DNA synthesis 20. However, evidence has demonstrated that the anticancer effect of GEM has been shown to be unsatisfactory, due to its chemoresistance 21. Therefore, it is urgent to develop new anticancer drugs or underlying resistant genes.

Lenvatinib, a new anticancer agent that acts by inhibiting the expression of VEGFR1-3, FGFR1-4, PDGFRα, SCF, RET and KIT, has been widely used for unresectable or metastatic HCC in China 5. Recent studies have reported that lenvatinib inhibited tumor growth and induced apoptosis in anaplastic thyroid cancer *in vitro* and *in vivo* by suppressing the expression of epidermal growth factor receptor, AKT, extracellular signal-regulated kinase 1/2 and VEGF-A 9. Wang *et al.* showed that lenvatinib promoted cell apoptosis in HK-1 cells by inducing endoplasmic reticulum stress 11. However, the effect of lenvatinib on GBC remains unclear. In the present study, lenvatinib exhibited anti-proliferation *in vitro* and *in vivo*. In addition, lenvatinib also induced apoptosis by increasing the expression of caspase-9. As expected, lenvatinib also regulated cell cycle arrest progression by decreasing the expression of CDK-2, CDK-4 and cyclin D1 in GBC cells. Here, the different result of cell cycle arrest progression between GBC-SD and NOZ cells may due to different kinds of GBC cells and cell biological characteristics.

A previous study demonstrated that lenvatinib inhibited migration and invasion in non-small cell lung cancer cells 22. He *et al.* also revealed that lenvatinib inhibited cell migration and invasion in HCC SMMC7721 and Hep3B cells by suppressing the expression of MMP-1, 2, 7, 9, 10 and 16 and increasing the expression of tissue inhibitor of metalloproteinases (TIMP)-1, 3 and 4 23. Lenvatinib significantly inhibited the migration and invasion of nasopharyngeal carcinoma HK-1 cells, as well as the expression of tumor angiogenesis-related proteins, such as VEGF and PDGF 11. The cell migration-inducing CEMIP gene was first reported in non-syndromic hearing loss in 2006 by He *et al.* 24. It was also reported that CEMIP has a regulatory effect on hyaluronan, which results in the occurrence of hyaluronic acid-rich organ diseases 25. Recent studies have demonstrated that the overexpression of CEMIP leads to tumor cell invasion, migration and carcinogenesis in many types of cancer 26. In the present study, lenvatinib inhibited cell migration and invasion by inhibiting the expression of MMP-2 and CEMIP in GBC-SD and NOZ cells.

Autophagy is a physiological cleaning process for mediating the degradation of intracellular components and damaged organelles. Accumulating evidences have shown that autophagy plays an important role in regulating biological processes, including cell proliferation, apoptosis, angiogenesis and chemoresistance 27. However, the role of lenvatinib in regulating autophagy remains unclear. In the present, we first reported the relationship between lenvatinib and autophagy. Treatment with lenvatinib significantly inhibited autophagy in GBC-SD and NOZ cells.

The PI3K/AKT pathway transmits signals from the cell membrane to the nucleus and plays an important role in oncogenesis. The PI3K/AKT pathway is known to play an important role in chemoresistance, and its activation has been shown to increase the chemoresistance of cancer cells 28. GEM is currently the first-line chemotherapeutic drug for bile tract and pancreatic cancer. Accumulating evidence has revealed that its benefit to cancer patients and level of and toxicity remain unsatisfactory. A previous study revealed that integrin β1 induced gemcitabine resistance in pancreatic cancer cells through activation of the PI3K signaling pathway 29. On the contrary, Wang *et al.* showed that LncRNA ab209630 enhanced the sensitivity of pancreatic ductal adenocarcinoma to gemcitabine by inhibiting the PI3K/AKT signaling pathway 30. In the present study, lenvatinib decreased the activation of PI3K and lenvatinib increased the sensitivity of GBC-SD and NOZ cells to gemcitabine through inhibiting the activation of PI3K. However, there are several limitations to the present study: lack of further machanism of how lenvatinib inhibited autophagy; the lack of a positive control (for example sorafenib, a known anticancer drug used to treat HCC).

In conclusion, the results of the present study revealed that lenvatinib inhibited cell growth and colony formation, induced cellular apoptosis by increasing the expression of cleaved caspase-9, and regulated the cell cycle at the G0/G1 or S phage. Furthermore, lenvatinib decreased cellular migration and invasion by inhibiting the expression of MMP-2 and CEMIP. Further investigation *in vivo* revealed that lenvatinib suppressed GBC cell growth by targeting p-AKT, which was consistent with the *in vitro* results. Mechanistically, lenvatinib promoted the sensitivity of GBC-SD and NOZ cells to gemcitabine by targeting the PI3K/AKT pathway. The mechanism of lenvatinib in mediating autophagy in GBC remains unclear, and it will be investigated further in our future study.
